# Exploring Sex Differences in Physical Activity Patterns Among Individuals with Schizophrenia Spectrum Disorders: Insights from the Diapason Project

**DOI:** 10.3390/jcm15051785

**Published:** 2026-02-27

**Authors:** Alessandra Martinelli, Elena Toffol, Giulia Moncalieri, Silvia Leone, Jacopo Santambrogio, Fabrizio Starace, Manuel Zamparini, Martina Carnevale, Giovanni de Girolamo, Stefano Calza

**Affiliations:** 1 Unit of Rehabilitation and Social Psychiatry, IRCCS Istituto Centro San Giovanni di Dio Fatebenefratelli, 25125 Brescia, Italy; 2 Unit of Epidemiological Psychiatry and Digital Mental Health, IRCCS Istituto Centro San Giovanni di Dio Fatebenefratelli, 25125 Brescia, Italy; 3 ASST Brianza Presidio Corberi e RSD Beato Papa Giovanni XXIII, 20871 Vimercate, Italy; 4 Department of Mental Health and Dependence, ASL Città di Torino, 10128 Turin, Italy; 5 Unit of Biostatistics and Bioinformatics, Department of Molecular and Translational Medicine, University of Brescia, 25121 Brescia, Italy

**Keywords:** schizophrenia spectrum disorder, physical activity, sex differences, accelerometer, sedentary lifestyle

## Abstract

**Background**: Schizophrenia spectrum disorders (SSD) affect females differently than males, yet there is limited research on Physical Activity (PA) levels and sex differences in patients with SSD. This study aimed at comparing PA levels between female and male SSD patients and controls. **Methods**: Altogether, 132 SSD residents and outpatients (48 females and 84 males) and 113 controls (46 females and 67 males) were assessed using standardised clinical tools. PA was monitored for seven consecutive days using a tri-axial ActiGraph GT9X accelerometer and quantified using the Euclidean Norm Minus One (ENMO) as an index of overall movement intensity. Descriptive and regression analyses were conducted. **Results**: Most patients were unemployed and overweight; males were less educated, less often divorced, smoking more, and using more antipsychotics than females (*p* < 0.05). Patients were less likely to be married, educated, employed, and had higher BMI and smoking rates than controls. Among patients, there were no significant sex differences in daily PA levels. In the control group, males showed slightly higher PA levels than females, although this difference did not reach statistical significance. Objective PA levels were not significantly associated with clinical outcomes in either female or male patients with SSD. **Conclusions**: Patients with SSD exhibited similarly low levels of objectively measured PA regardless of sex, suggesting a “flattening” phenomenon of sex differences in PA. These findings highlight the need for interventions aimed at promoting PA in individuals with SSD and support further research to identify factors influencing PA engagement across sexes.

## 1. Introduction

Schizophrenia spectrum disorders (SSD) are chronic and debilitating mental disorders characterised by severe disruption of thought processes, emotions, and perceptions. Individuals diagnosed with SSD often experience significant impairments in various aspects of their lives, including physical health and well-being [1,2], and have high rates of somatic comorbidities, such as diabetes, cardiovascular disorders, and metabolic syndrome [3,4].

Several sex differences have been found in patients with SSD. For example, compared to female patients, male patients are likely to be younger at onset; show more negative symptoms, such as social withdrawal, apathy, and diminished emotional expression; have a more chronic and severe course of illness, with increased hospitalisations and poorer functional outcomes; and show greater cognitive impairments, with deficits in executive functioning and verbal memory. On the other hand, female patients tend to be older at onset; show more affective symptoms such as depression, anxiety, and emotional dysregulation; enjoy longer periods of remission and better overall functioning; and have better verbal abilities and more favourable response to antipsychotics, but are at a higher risk of side effects such as weight gain and metabolic disturbances [5,6,7,8,9,10,11,12,13].

Previous research indicates that individuals with SSD tend to spend a significant amount of their daily time in sedentary behaviour and engage in minimal activities [14]. More inactivity is associated with higher risks of cardiovascular diseases and mortality rates [15].

In this regard, Physical Activity (PA), an essential component of a healthy lifestyle, has been recognised as a crucial factor in maintaining and improving overall health [16,17]. While the importance of PA for individuals with SSD is well-established [16,17], there is limited research specifically focusing on sex differences within this population [18]. In particular, the comparison between PA levels in female and male patients with SSD and healthy controls remains largely unexplored.

Accelerometer-based biosensors, widely recognised as valid and reliable tools for objectively measuring PA both longitudinally and in ecologically valid contexts, provide a detailed and accurate assessment of individuals’ movement patterns. This objective approach allows for a more comprehensive understanding of the differences in PA patterns across various populations [19,20,21,22].

Thus, the current study aims to address the research gap described above by assessing PA levels with accelerometers in female and male patients with SSD and paired healthy controls. By incorporating both sex and diagnostic factors into the analysis, we seek to explore potential differences in PA patterns, elucidate underlying factors contributing to these differences, and identify areas for intervention.

The existing literature [19,23,24] suggests that female individuals with SSD face unique challenges and barriers to engaging in PA compared to their male counterparts and healthy female controls [25]. Therefore, we hypothesise that female individuals with SSD may exhibit lower levels of PA compared to healthy female controls. Additionally, we expect to observe disparities between male and female patients within the SSD group, with male patients showing higher PA levels. We also expect that male control participants will exhibit higher levels of PA than male patients.

This comparative analysis aims to contribute to the growing body of knowledge on the PA profiles of individuals with SSD, while specifically focusing on sex differences. By highlighting potential disparities, findings from this prospective cohort study may inform the development of sex-specific interventions, tailored exercise programmes, and effective strategies to promote PA and physical health outcomes among patients with SSD. Ultimately, such initiatives have the potential to improve overall physical and mental health outcomes and enhance the quality of life for individuals with SSD.

## 2. Material and Methods

### 2.1. Study Setting: DiAPAson

In Italy, individuals with SSD receive treatment from 123 Departments of Mental Health (DMHs). These DMHs offer various types of care, including outpatient, hospital, and residential facilities (RFs). RFs are specifically designed to support and supervise individuals with relevant psychosocial impairments. These individuals may struggle to independently manage their symptoms and daily activities [26,27,28]. This cohort study was conducted in a subsample of sites involved in the Italian national DiAPAson project: these include seven DMHs, one clinical research centre (IRCCS) and two RFs. Detailed information about the project can be found in the study protocol [29]. The DMHs enlisted both outpatients and residents, whereas RFs exclusively recruited residents. Overall, the project enrolled a total of 98 RFs, each recruiting an average of 3.3 (±2.6) residents, which corresponds to approximately 27% of patients within each RF.

### 2.2. DiAPAson Procedure and Participants

At each study centre, clinicians responsible for the treatment invited their patients to participate in the study and recruited healthy controls matched for age and sex with patients. Outpatients were community-dwelling patients with SSD who were approached consecutively at the outpatient units for potential participation until the recruitment target was achieved. Residents were recruited using an alphabetical list of patients with SSD present on an index day; based on this list, they were consecutively invited to participate in this study.

The inclusion criteria for the study were (i) a DSM-5 SSD diagnosis [30]; (ii) 20–55 years of age; and (iii) proficiency in speaking and writing Italian. Patients who were unable to give informed consent, reported severe cognitive deficits (indicated by a Mini-Mental State Examination corrected score below 24), had a recent (last six months) diagnosis of substance use disorder according to DSM-5 criteria [30], a history of clinically significant head injury, or cerebrovascular/neurological disease were excluded from the study.

**Figure 1:**
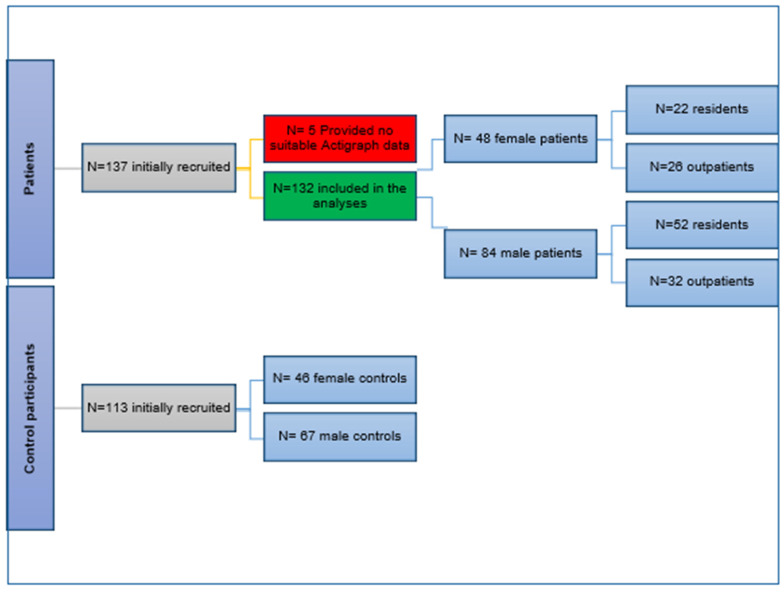
Flowchart of sampling selection.

A total of 250 participants were recruited from October 2020 to October 2021: 137 (N = 79 residents and N = 58 outpatients) met the criteria required for SSD diagnosis and 113 were healthy controls. To be included in the analyses, each participant had to have at least 4 valid accelerometer monitoring days and at least 10 valid hours of wearing time for each day. Five participants were excluded from the analyses since they had less than four valid monitoring days of actigraphy data. The data recruitment process is illustrated in the flow chart in Figure 1. As a result, the final sample for analysis consisted of 132 patients and 113 healthy controls. Among the participants, there were 48 female patients with SSD and 84 male patients with SSD, and they were all matched with 46 female controls and 67 male controls. As a token of appreciation, outpatients with SSD and controls received €25.00 each during the debriefing session to cover their travel expenses.

### 2.3. Assessments

Participants included in the DiAPAson project underwent a comprehensive evaluation using various standardised tools. Detailed information regarding the assessment can be found in the study protocol [29] and Supplementary Table S1. The assessment process involved a combination of clinician-administered instruments/measures and self-reported questionnaires, with the assistance of Research Assistants for the latter.

### 2.4. Assessment of PA

All participants in the study were given guidance in wearing an ActiGraph GT9X Link (manufactured by ActiGraph, Pensacola, FL, USA), a validated triaxial accelerometer worn on their non-dominant wrist continuously for seven consecutive days. This device was used to monitor PA levels and track patterns of sleep–wake cycles.

### 2.5. Statistical Analysis

Data were described using mean and standard deviation (SD) or median and interquartile range for quantitative variables, while qualitative variables were presented as counts and percentages. Sociodemographic and clinical variables were compared among patients and controls using the χ^2^ test, with *p*-values computed using Monte Carlo simulation (B = 200) for categorical variables and the analysis of variance for continuous variables.

Raw acceleration data were processed using the GGIR package [31]. Following auto-calibration, Euclidean Norm Minus One (ENMO) was derived from raw triaxial acceleration signals (ActiGraph GT9X Link (manufactured by ActiGraph, Pensacola, FL, USA) as an index of overall movement intensity, representing the vector magnitude of dynamic acceleration after removal of the gravitational component [32]. Data were analysed using 60 s epochs, with no imputation of missing ENMO values. Participants were included if they provided at least four valid days with ≥10 h of wear time per day.

Bedtime and sleep periods were estimated using a combined algorithm. First, the Cole–Kripke algorithm classified each 60 s epoch as either bedrest or active. Subsequently, the Tudor–Locke algorithm was applied to identify continuous sleep periods, using the following criteria: minimum sleep time = 5 min, minimum awake time = 15 min, and minimum sleep period length = 120 min.

Linear regression models were used to explore the relationships between psychiatric severity/levels of functioning (Brief Psychiatric Rating Scale—BPRS, Brief Negative Symptom Scale—BNSS, Specific Levels of Functioning Scale—SLOF scales) and daily mean PA, adjusted for daily % of wearing time, number of cigarettes, season of measurement and number of antipsychotic drugs. An interaction term between sex and ENMO was included to estimate sex-specific effects. CIs for BPRS, BNSS, and SLOF estimates were computed via bootstrap due to highly skewed distributions. All tests were two-sided and assumed a significance level of 5%. To control for multiple testing, Holm–Bonferroni correction was applied. Effect sizes were interpreted alongside statistical significance to mitigate potential Type II error. Confidence intervals for BPRS, BNSS, and SLOF estimates were computed using nonparametric bootstrap resampling (1000 iterations) due to highly skewed distributions. All the analyses were performed using R version 4.3.1.

## 3. Results

### 3.1. Sociodemographic and Clinical Characteristics of Female and Male Patients with SSD and Female and Male Controls

As shown in Table 1, most female and male patients in the sample were between 43 and 55 years old (F = 52.1%; M = 50.0%) and unemployed (F = 64.6%; M = 58.3%). Female patients exhibited a significantly higher divorce rate (*p* = 0.006) and a higher level of education compared to male patients (*p* = 0.003).

There were no significant differences between male and female patients in the Charlson Comorbidity Index (*p* = 0.600) and body mass index (BMI; *p* > 0.900). On average, all patients with SSD were overweight [(BMI F: mean = 27.2 (SD = 6.1); M: mean = 27.2 (SD = 5.5)]. Males had a significantly higher weight (*p* < 0.001) and smoked more cigarettes than female patients (*p* = 0.045). There were no significant sex differences in self-perception of disability and clinical characteristics related to SSD, except for a higher use of antipsychotics in male patients (*p* = 0.036) (see Table 2).

As shown in Table 1, there were no differences in sociodemographic characteristics between female and male healthy controls, while male controls had significantly higher BMI (*p* = 0.012), weight (*p* < 0.001) and waist circumference (*p* < 0.001) than the female controls.

In a comparison between patients and controls’ characteristics stratified by sex, patients were significantly less likely to be married/cohabitating (*p* < 0.001), educated (*p* < 0.001), and employed (*p* < 0.001) compared to their sex-paired controls. Weight, BMI, waist circumference, and cigarette consumption of patients were significantly higher than those of controls (Supplementary Table S2).

### 3.2. Comparison of Daily PA Levels in Patients with SSD and Controls by Sex

Table 3 reports the comparison of daily PA between patients with SSD and controls, separately for females and males. In patients with SSD, both females and males showed a mean ENMO of 3.5 (SD 0.5; *p* = 0.9). In the control group, females had a mean ENMO of 3.8 (SD 0.4), whereas males had a mean ENMO of 3.9 (SD 0.4). This difference showed a trend toward significance but did not reach the conventional threshold for statistical significance (*p* = 0.058).

**Figure 2:**
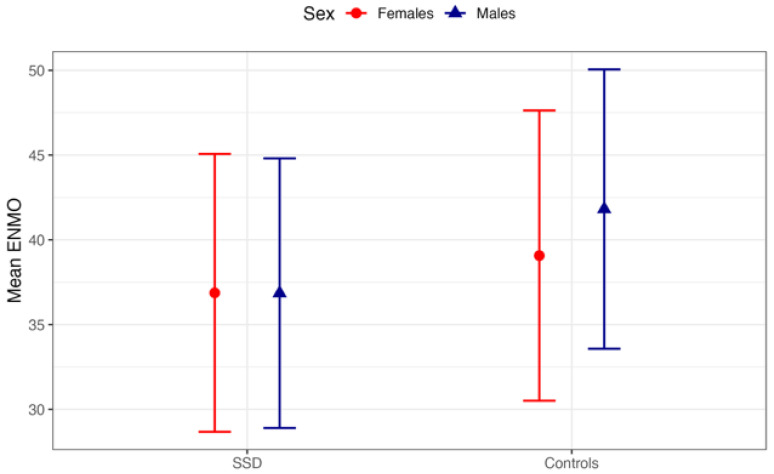
Differences in PA (mean ENMO) between patients with SSD and control participants by sex.

These patterns are visually represented in Figure 2, which shows overlapping ENMO values for females and males in the SSD group and a modest separation between sexes in the control group, with higher values observed in males.

### 3.3. Relationship Between PA Levels and Clinical Outcomes in Female and Male Patients with SSD

The associations between PA and clinical outcomes in patients with SSD, stratified by sex, are reported in Table 4. ENMO was not significantly associated with global psychopathology (BPRS), negative symptoms (BNSS), or functioning (SLOF) in either females or males. In females, ENMO showed non-significant negative associations with BPRS (β = −1.23, 95% CI −4.32 to 1.29), BNSS (β = −0.31, 95% CI −3.83 to 3.57), and SLOF (β = −2.24, 95% CI −7.67 to 2.24). Similarly, in males, ENMO was not significantly associated with BPRS (β = −0.66, 95% CI −2.61 to 1.31), BNSS (β = −072, 95% CI −2.93 to 1.63), or SLOF (β = 2.35, 95% CI −0.34 to 4.90).

## 4. Discussion

This study provides valuable insights into the PA of individuals with SSD, focusing specifically on sex differences. The analysis aims to explore and highlight any sex-based differences in PA among patients with SSD and controls according to sex. We utilised accelerometer-based biosensors to objectively measure PA, allowing for a detailed and accurate assessment of movement patterns.

Contrary to our initial expectations, we found that male and female patients with SSD showed similar profiles of PA. Sex does not appear to influence movement levels in this clinical group. In the control group, males tend to be slightly more active than females, but this difference represents only a trend and cannot be considered statistically significant. These findings suggest that SSD may reduce sex differences in PA, although direct evidence in the literature is limited. This apparent “flattening” could be driven by common disease-related factors affecting both sexes, such as negative symptoms [33] and “side-effects of antipsychotic medication in general (e.g., motor, sedative, or metabolic side-effects) which are often reported barriers to PA” [34]. Importantly, the use of psychotropic medication, particularly antipsychotics, may influence PA through sedation, extrapyramidal symptoms, and metabolic burden, regardless of whether treatment is administered as monotherapy or polypharmacy. Of note, in our study male patients were more likely than female patients to use antipsychotics. Comorbidities, such as diabetes, may further contribute to reduced PA in both males and females [35]. Overall, our results are consistent with previous studies documenting high sedentary behaviour and low PA among individuals with SSD [36,37,38] and highlight potential health risks associated with sedentariness.

Moreover, compared to controls, less patients were married, educated, employed, and had higher BMI and smoking rates. These findings suggest that the flattened PA among male and female patients may be closely related to these sociodemographic, clinical challenges and other lifestyle habits. Unemployment and singlehood can lead to social isolation [7,39,40], reducing opportunities and motivation for engaging in PA. Overweight status and higher BMI can contribute to physical discomfort, health issues [41], and worse mental health [42], further discouraging activity. Higher smoking rates might also correlate with lower energy levels, higher heart rates and respiratory rates, indicating reduced exercise capacity [43]. In addition, longer duration of disorder and chronic exposure to psychotropic treatments may further compound these barriers, progressively limiting engagement in PA over time. This indicates that PA may play a relevant role in enhancing the overall well-being of individuals with SSD.

Objective PA levels were not significantly associated with symptom severity or functional outcomes in either sex within the SSD group. Taken together, these findings suggest that sex differences in PA are absent in patients with SSD, both at the descriptive level and in terms of clinical correlates, whereas a weak sex-related pattern in PA is detectable in the control population. These findings suggest that for patients with SSD, the lack of association between PA and clinical outcomes may indicate a need for more targeted interventions irrespective of patients’ sex to promote PA and maximise its health benefits. Interventions aimed to increase PA among patients with SSD should target specific barriers to PA.

### Strengths and Limitations

This study has some limitations. First, this study was conducted during the SARS-CoV-2 pandemic, which influenced daily clinical practice and routine activities in both patient living settings, as well as in healthy controls. Nevertheless, as the recruitment of patients and controls occurred concurrently, this alteration probably impacted both study groups. Additionally, the diagnosis of SSD relied solely on medical records due to financial and logistical constraints. The assessment of activity at the wrist tends to overstate PA and faces challenges in distinguishing between sitting and standing.

Furthermore, detailed information on antipsychotic treatment characteristics (e.g., dosage, monotherapy versus polypharmacy, treatment duration) and duration of illness was not available for all participants, limiting a more granular analysis of their potential impact on PA levels. Additionally, the cross-sectional design of the study limits the interpretation of the associations between PA levels and psychological outcomes in terms of directionality and causality. Finally, the lack of qualitative data hinders a deeper understanding of the differences in the ways male and female patients engage in PA, which is important for explaining the varying results observed in patients’ PA levels by sex and setting.

Even with the above limitations, strengths of the study include an objective and detailed assessment of PA using accelerometers and a relatively large sample size with data collected in multiple sites across Italy, leading to good geographical generalisability. Additionally, the reliability of psychopathological profiles and psychosocial functioning data is guaranteed using multiple, widely established assessment tools [44].

## 5. Conclusions

In conclusion, our findings indicate that SSD may in fact have a negative impact on PA levels among both outpatients and residents, irrespective of their sex. As such, they carry important clinical implications, as they indicate that promoting PA can be beneficial in terms of well-being and outcomes, and thus, it should be included as part of treatment approaches. The promotion of specific programmes for patients with SSD has been recently recommended by expert centres for SSD [40]. Not highlighting sex differences, our prospective cohort study further suggests the need for sex-specific research to understand which interventions and tailored exercise programmes are adequate to promote PA in patients with SSD according to sex. Such initiatives have the potential to enhance physical and psychosocial health outcomes for individuals with SSD of different sex. In this context, structured and tailored Physical Activity programmes may represent a feasible and effective tool to be integrated into the multidisciplinary care of patients with SSD. Nevertheless, further explorations are needed to deepen our comprehension of the nuanced relationship between SSD, sex and PA.

## Figures and Tables

**Table 1:** Sociodemographic characteristics of patients with SSD and control participants.

	Patients with SSD				Control Participants			
	Female	Male			Female	Male		
	N = 48 (36.4%)	N = 84 (63.6%)	*p*-Value ^1^	q-Value ^2^	N = 46 (41.0%)	N = 67 (59.0%)	*p*-Value ^1^	q-Value ^2^
**Age, n (%)**			0.600	>0.900			>0.900	>0.900
20–30	10 (20.8%)	16 (19.0%)			8 (17.0%)	11 (16.4%)		
31–42	12 (25.0%)	26 (31.0%)			15 (33.0%)	22 (32.8%)		
43–55	25 (52.1%)	42 (50.0%)			23 (50.0%)	33 (49.3%)		
>55	1 (2.1%)	0 (0.0%)			0 (0.0%)	1 (1.5%)		
**Marital status, n (%)**			**0.006**	0.100			>0.900	>0.900
Single	34 (71.0%)	75 (89.3%)			13 (28.3%)	16 (24.0%)		
Married or cohabiting	5 (10.0%)	6 (7.1%)			30 (65.2%)	47 (70.0%)		
Divorced or widowed	9 (19.0%)	3 (3.6%)			3 (6.5%)	4 (6.0%)		
** Education (years)**			**0.003**	0.055			0.200	>0.900
Mean (SD)	13.0 (3.3)	11.4 (2.8)			17.3 (4.8)	16.0 (4.9)		
Median (Minimum; Maximum)	13.0 (7.0; 21.0)	12.0 (2.0; 18.0)			17.0 (8.0; 26.0)	16.0 (6.0; 27.0)		
**Working status, n (%)**			0.800	>0.900			>0.900	>0.900
Working	14 (29.2%)	28 (33.33%)			43 (93.5%)	61 (91.0%)		
Studying	3 (6.2%)	7 (8.33%)			3 (6.5%)	5 (7.5%)		
Not working	31 (64.6%)	49 (58.33%)			0 (0.0%)	1 (1.5%)		
**Charlson Comorbidity Index**			0.600	>0.900			0.400	>0.900
Mean (SD)	0.9 (1.4)	0.7 (1.3)			0.6 (0.9)	0.4 (0.7)		
Median (Minimum; Maximum)	0.0 (0.0; 6.0)	0.0 (0.0; 8.0)			0.0 (0.0; 4.0)	0.0 (0.0; 3.0)		
**Body Mass Index**			>0.900	>0.900			**0.012**	**0.083**
Mean (SD)	27.2 (6.1)	27.2 (5.5)			23.1 (3.9)	24.9 (3.5)		
Median (Minimum; Maximum)	26.6 (17.7; 44.8)	26.2 (16.6; 44.3)			21.8 (18.8; 34.7)	24.6 (17.7; 35.5)		
**Weight (kg)**			**<0.001**	**0.015**			**<0.001**	**<0.001**
Mean (SD)	73.7 (16.0)	85.2 (19.7)			62.4 (9.7)	79.5 (13.3)		
Median (Minimum; Maximum)	75.5 (42.0; 109.0)	81.8 (50.0; 150.0)			59.3 (44.0; 84.0)	76.0 (55.0; 115.0)		
**Waist Circumference (cm)**			0.140	>0.900			**<0.001**	**<0.001**
Mean (SD)	97.5 (15.2)	101.8 (16.0)			82.3 (10.7)	92.6 (11.7)		
Median (Minimum; Maximum)	97.0 (71.0; 126.0)	100.5 (78.0; 155.0)			80.0 (60.0; 103.0)	91.0 (59.0; 123.0)		
**Smoking (cigarettes per day)**			**0.045**	0.600			0.500	**<0.001**
Mean (SD)	6.6 (10.9)	10.5 (10.7)			2.2 (4.5)	1.7 (4.6)		
Median (Minimum; Maximum)	0.0 (0.0; 40.0)	10.0 (0.0; 40.0)			0.0 (0.0; 17.0)	0.0 (0.0; 22.0)		

Bold values indicate statistical significance at the *p* < 0.05 level. ^1^ Pearson’s Chi-squared test with simulated *p*-value (based on 2000 replicates); one-way analysis of means. ^2^ Holm–Bonferroni correction for multiple testing.

**Table 2:** Clinical characteristics of female and male patients with SSD.

	FEMALE N = 48 (36.4%)	MALE N = 84 (63.6%)	*p*-Value ^1^	q-Value ^2^
**Living, n (%)**			0.110	>0.900
Residential facility	22 (46.0%)	52 (62.0%)		
Private accommodation (outcare)	26 (54.0%)	32 (38.0%)		
** Illness duration (years)**			0.368	>0.900
Mean (SD)	17.0 (8.8)	18.7 (10.8)		
** Lifetime duration of psychiatric hospitalisation (years), n (%)**			0.400	>0.900
<1 years	267(56.3%)	36 (42.8%)		
1–5 years	10 (21.3%)	22 (26.2%)		
>5 years	11 (23.4%)	26 (31.0%)		
** Brief Psychiatric Rating Scale (BPRS)**			0.913	>0.900
Mean (SD)	45.9 (11.9)	45.5 (13.0)		
** Brief Negative Symptom Scale (BNSS)**			0.765	>0.900
Mean (SD)	21.0 (15.9)	21.8 (14.1)		
** Specific Levels of Functioning Scale (SLOF)**			0.500	>0.900
Mean (SD)	183.3 (20.8)	180.8 (20.2)		
** WHO Disability Assessment Schedule (WHODAS 2.0)**			0.500	>0.900
Mean (SD)	11.9 (7.6)	10.9 (8.1)		
** Aps drugs**			**0.007**	**0.110**
Mean (SD)	1.9 (1.0)	2.5 (1.3)		
**Non-Aps drugs**			0.400	>0.900
Mean (SD)	0.6 (0.7)	0.5 (0.6)		

Aps = antipsychotics. Bold values indicate statistical significance at the *p* < 0.05 level. ^1^ Pearson’s Chi-squared test with simulated *p*-value (based on 2000 replicates); one-way analysis of means. ^2^ Holm–Bonferroni correction for multiple testing.

**Table 3:** Differences in PA in patients with SSD and control participants according to sex.

ENMO-SSD				
Variables	Female N = 48 (36%)	Male N = 84 (64%)	*p*-Value ^1^	q-Value ^2^
**ENMO**			0.9	>0.900
Mean (SD)	3.5 (0.5)	3.5 (0.5)		
Median (Min; Max)	3.5 (2.1; 4.5)	3.5 (2.4; 4.6)		
**ENMO-Controls**				
**Variables**	**Female** **N = 46 (41%)**	**Male** **N = 67 (59%)**	***p*** **-Value ^1^**	**q-Value ^2^**
**ENMO**			0.058	>0.900
Mean (SD)	3.8 (0.4)	3.9 (0.4)		
Median (Min; Max)	3.8 (2.6; 4.6)	4.0 (2.8; 4.6)		

ENMO = Euclidean Norm Minus One; SSD = schizophrenia spectrum disorder. ^1^ One-way analysis of means. ^2^ Holm–Bonferroni correction for multiple testing.

**Table 4:** Association between PA (ENMO) and clinical outcomes between patients with SSD according to sex.

PA	BPRS (F)	BPRS (M)	BNSS (F)	BNSS (M)	SLOF (F)	SLOF (M)	*p*-Value	*p*-Value	*p*-Value
**ENMO**	−1.2251 (−4.3234; 1.2944)	−0.6635 (−2.6163; 1.31)	−0.3062 (−3.8378; 3.5697)	−0.7224 (−2.938; 1.628)	−2.2422 (−7.6866; 2.2415)	2.3512 (−0.3468; 4.9041)	0.4539	0.8737	0.3878

All models were adjusted for daily % of wearing time, number of cigarettes, season of measurement, and antipsychotic drugs. Average group estimates are relative to 90% daily wearing time; ENMO = Euclidean Norm Minus One; β coefficients (95% CI) stratified by sex.

## Data Availability

Dataset referring to this manuscript is published with restricted access on Zenodo platform and accessible at this link: https://doi.org/10.5281/zenodo.10119250.

## Supplementary Materials

The following supporting information can be downloaded at: https://www.mdpi.com/article/10.3390/jcm15051785/s1. Table S1: Clinician-administered instruments or measures and self-reported questionnaires; Table S2: Comparison of sociodemographic characteristics of patients with SSD and control participants according to sex.
