# Supplementary material for: Exploring Sex Differences in Physical Activity Patterns Among Individuals with Schizophrenia Spectrum Disorders: Insights from the Diapason Project
Source: J Clin Med. 2026 Feb 27;15(5):1785. doi: 10.3390/jcm15051785 (PMC12986268; doi:10.3390/jcm15051785)
Supplement: Supplementary file 1 [file jcm-15-01785-s001.zip › jcm-4139930-supplementary.pdf]

Supplementary Table S1 Clinician-administered instruments or measures and self-reported questionnaires.

Instrument/measure/questionnaire	Description	Patients	Controls
**Charlson Comorbidity Index**<br>• Charlson ME, Pompei P, Ales KL, MacKenzie CR. A new method of classifying prognostic comorbidity in longitudinal studies: development and validation. J Chronic Dis 1987;40(5):373-383.	It measures health status/risk of death, considering 19 medical conditions (16 diseases of which 3 are stratified according to severity) that are given a weight ranging from 1 to 6 with a final score ranging from 0 to 33.	X	X
**Body mass index (BMI)**<br>• https://www.nhs.uk/common-health-questions/lifestyle/what-is-the-body-mass-index-bmi/	It is obtained from height and weight (BMI = kg/m2).<br>Normal weight (BMI < 25), Overweight (25 ≤ BMI < 30), Obese (BMI ≥ 30).	X	X
**Waist circumference**<br>• Browning LM, Mugridge O, Dixon AK, Aitken SW, Prentice AM, Jebb SA. Measuring abdominal adipose tissue: comparison of simpler methods with MRI. Obes Facts. 2011;4(1):9-15. doi: 10.1159/000324546.	This measurement is a surrogate for abdominal adipose tissue. The participant stands upright, and if more convenient, they can use a low platform for measurement. The abdomen should be uncovered, and the tape measure, held parallel to the floor, is placed just above the iliac crests. Ensure the tape is taut but not compressing the skin. For waist circumference, measure the narrowest part just above the iliac crest or at the navel if the former is not visible.	X	X
**Brief Psychiatric Rating Scale (BPRS)**<br>• Overall JE, Gorham DR. The Brief Psychiatric Rating Scale. Psychological Reports 1962;10:799-812.<br>• Morosini P, Casacchia M, Ventura J. Brief Psychiatric Rating Scale (BPRS): versione 4.0 ampliata : manuale di istruzioni : [scale, definizioni operative dei livelli di gravità dei sintomi e domande dell'intervista]. ; 1995.	The 24-item BPRS is used to evaluate symptom severity. The BPRS items are rated on a 7-point Likert scale, ranging from 1 (no symptoms) to 7 (extremely severe symptoms). The items are categorized into five domains (depression/anxiety, excitement, positive symptoms, negative symptoms, and cognitive symptoms). Higher mean scores indicate a higher level of symptomatology.	X	-
**Brief Negative Symptom Scale (BNSS)**<br>• Strauss GP, Keller WR, Buchanan RW, Gold JM, Fischer BA, McMahon RP, et al. Next-generation negative symptom assessment for clinical trials: validation of the Brief Negative Symptom Scale. Schizophr Res 2012 Dec;142(1-3):88-92.<br>• Mucci A, Galderisi S, Merlotti E, Rossi A, Rocca P, Bucci P, et al. The Brief Negative Symptom Scale (BNSS): Independent validation in a large sample of Italian patients with schizophrenia. Eur Psychiatry 2015 Jul;30(5):641-647.	It is employed to assess the severity of negative symptoms with items rated on a 6-point Likert scale ranging from 0 (no symptoms) to 6 (severe symptoms). The items evaluate blunted affect, alogia, asociality, anhedonia, and avolition. Higher mean scores indicate a higher level of symptomatology.	X	-
**Specific Levels of Functioning Scale (SLOF)**<br>• Montemagni C, Rocca P, Mucci A, Galderisi A, Maj M. Italian version of the 'Specific Level of Functioning'. Journal of Psychopathology 2015;21(3):287-296.	It evaluates psychosocial functioning, assessing the level of support required by participants to perform various tasks. The scale consists of 43 items, each rated on a 5-point Likert scale ranging from 1 (no support needed) to 5 (extensive support needed). The overall total score on the SLOF ranges from 43 to 215, with a higher total score indicating higher levels of psychosocial functioning.	X	-

• Rocca P, Galderisi S, Rossi A, Bertolino A, Rucci P, Gibertoni D, et al. Disorganization and real-world functioning in schizophrenia: Results from the multicenter study of the Italian Network for Research on Psychoses. Schizophr Res 2018 Nov;201:105-112.			
**WHO Disability Assessment Schedule (WHODAS 2.0)**<br>• World Health Organization, 2012. Measuring health and disability: manual for WHO Disability Assessment Schedule (WHODAS 2.0), World Health Organization, 2010, Geneva.	It is a standardized self-report instrument measuring functioning and disability over the past 30 days. The 12-item version was used, with each item rated on a 5-point Likert scale (fc) ranging from no difficulty to extreme difficulty/cannot do, with higher scores indicating greater disability.	X	-

X indicates the instances when the instrument/measure/questionnaire was employed with either patients diagnosed with SSD or control participants.

Supplementary Table S2 Comparison of sociodemographic characteristics of patients with SSD and control participants according to sex.

	FEMALE			MALE		
**VARIABLES**	**Patients with SSD N=48 (52.1%)**	**Control participants N=46 (48.9%)**	**p-value***	**Patients with SSD N=84 (55.6%)**	**Control participants N=67 (44.4%)**	**p-value***
**Age, n (%)***			0.730			0.800
20-30	10 (20.8%)	8 (17.0%)		16 (19.0%)	11 (16.4%)	
31-42	12 (25.0%)	15 (33.0%)		26 (31.0%)	22 (32.8%)	
43-55	25 (52.1%)	23 (50.0%)		42 (50.0%)	33 (49.3%)	
>55	1 (2.1%)	0 (0.0%)		0 (0.0%)	1 (1.5%)	
**Marital status, n (%)**			**<0.001**			**<0.001**
Single	34 (71.0%)	13 (28.3%)		75 (89.3%)	16 (24.0%)	
Married or cohabiting	5 (10.0%)	30 (65.2%)		6 (7.1%)	47 (70.0%)	
Divorced or widowed	9 (19.0%)	3 (6.5%)		3 (3.6%)	4 (6.0%)	
**Education years**			**<0.001**			**<0.001**
Mean (SD)	13.0 (3.3)	17.3 (4.8)		11.4 (2.8)	16 (4.9)	
Median (Min–Max)	13.0 (7.0; 21.0)	17.0 (8.0; 26.0)		12 (2.0; 18.0)	16.0 (6.0; 27.0)	
**Working status, n (%)**			**<0.001**			**<0.001**
Working	14 (29.2%)	43 (93.5%)		28 (33.3%)	61 (91.0%)	
Studying	3 (6.2%)	3 (6.5%)		7 (8.3%)	5 (7.5%)	
Not working	31 (64.6%)	0 (0.0%)		49 (58.4%)	1 (1.5%)	
**Charlson Comorbidity Index***			0.083			0.093
Mean (SD)	0.9 (1.4)	0.6 (0.9)		0.7 (1.3)	0.4 (0.7)	
Median (Min–Max)	0.0 (0.0; 6.0)	0.0 (0.0; 4.0)		0.0 (0.0; 8.0)	0.0 (0.0; 3.0)	
**Weight (Kg)**			**<0.001**			**0.045**
Mean (SD)	73.7 (16.0)	62.4 (9.7)		85.2 (19.7)	79.5 (13.3)	
Median (Min–Max)	75.5 (42.0; 109.0)	59.3 (44.0; 84.0)		81.8 (50.0; 150.0)	76.0 (55.0; 115.0)	
**Body Mass Index (BMI)**			**<0.001**			**0.004**
Mean (SD)	27.2 (6.1)	23.1 (3.9)		27.2 (5.5)	24.9 (3.5)	
Median (Min–Max)	26.6 (17.7; 44.8)	21.8 (18.8; 34.7)		26.2 (16.6-44.3)	24.6 (17.7; 35.5)	
**Waist Circumference (cm)**			**<0.001**			**<0.001**
Mean (SD)	97.5 (15.2)	82.3 (10.7)		101.8 (16.0)	92.6 (11.7)	
Median (Minimum; Maximum)	97.0 (71.0; 126.0)	80.0 (60.0; 103.0)		100.5 (78.0; 155.0)	91.0 (59.0; 123.0)	
**Smoking (cig./day)***			**0.013**			**<0.001**
Mean (SD)	6.6 (10.9)	2.2 (4.5)		10.5 (10.7)	1.7 (4.6)	
Median (Min–Max)	0.0 (0.0; 40.0)	0.0 (0.0; 17.0)	-	10.0 (0.0; 40.0)	0.0 (0.0; 22.0)	

*Pearson's Chi-squared test with simulated p-value (based on 2000 replicates).
Bold values indicate statistical significance at the $p < 0.05$ level.
